# Primary Intraosseous Squamous Cell Carcinoma of the Mandible: A Rare and Challenging Case

**DOI:** 10.7759/cureus.85690

**Published:** 2025-06-10

**Authors:** Sanjay Byakodi, Mushtak Khan, Jaydeep Nilkanthrao Pol, Girish Anandrao Kadkol, Gautam Purohit, Devesh Agiwal

**Affiliations:** 1 Department of Oral and Maxillofacial Surgery, Bharati Vidyapeeth (Deemed to be University) Dental College and Hospital, Sangli, IND; 2 Department of Surgical Pathology, Mahatma Gandhi Cancer Hospital, Miraj, IND; 3 Department of Head and Neck Oncosurgery, Bharati Vidyapeeth (Deemed to be University) Medical College and Hospital, Sangli, IND

**Keywords:** dentistry, lesion, rare, resection, squamous cell carcinoma

## Abstract

Primary intraosseous squamous cell carcinoma (PIOSCC) is a rare cancer that arises within the jaws, often linked to odontogenic cysts or tumors. Its prevalence is about 1-2.5% of all odontogenic tumors and presents a diagnostic challenge due to its similarity to other jaw lesions. A 46-year-old woman complained of persistent pain in the left back region of the lower jaw, numbness over the left side of the lower lip, and reduced mouth opening. The doctor revised the initial diagnosis of periapical infection due to ongoing symptoms and a non-healing socket. Radiological imaging revealed an osteolytic lesion in the left ramus of the mandible, prompting further investigation. A biopsy confirmed the diagnosis of IOSCC following histopathological examination after segmental mandibulectomy. The final diagnosis was primary intraosseous well-differentiated squamous cell carcinoma of the left mandible. PIOSCC, even though it is uncommon, should be included in the list of possible diagnoses for jaw problems, especially those that have unclear, bone-destroying X-ray features. Symptoms such as pain, swelling, and sensory disturbances, along with radiologic findings, may suggest a malignant odontogenic tumor. Histological evaluation is crucial for differentiation from other odontogenic tumors, including ameloblastic carcinoma. Radical surgery, often combined with neck dissection, is the management of choice. Postoperative radiotherapy or chemotherapy may be considered, though their role remains unclear. The five-year survival rate with PIOSCC ranges from 30% to 46%, indicating a typically dismal prognosis. PIOSCC is an uncommon, aggressive tumor with a poor prognosis. Early diagnosis and accurate histopathological examination are essential to differentiate it from other odontogenic carcinomas and improve patient outcomes.

## Introduction

Primary intraosseous squamous cell carcinoma (PIOSCC) is a rare malignant tumor of the jaws, not initially connected to the oral mucosa. It most likely arises from an odontogenic cyst or tumor or from the remains of odontogenic epithelium. Morrison and Deeley point out that Loos was the first to describe it in 1913. After Wills changed the nomenclature in 1948 to "intra-alveolar epidermoid carcinoma," Shear changed it to "primary intra-alveolar epidermoid carcinoma." The 2005 WHO classification changed the term "primary intraosseous carcinoma" (PIOC) to "primary intraosseous squamous cell carcinoma" (PIOSCC) [1].

The WHO divided PIOSCC into three categories: solid-type carcinoma, carcinoma that comes from a keratocystic odontogenic tumor, and carcinoma that starts from an odontogenic cyst [2]. PIOSCC accounts for about 12% of all oral cancers. Odontogenic cysts, such as keratocystic odontogenic tumors and dentigerous cysts, cause the majority of these cases. Residual periapical cysts are the source of a small percentage of cases. Pain, swelling of the jaw, and sensory abnormalities are typical symptoms. Other primary tumors need to be checked out before PIOSCC is diagnosed. Histological results are frequently inconclusive in terms of diagnosis [3].

Predilection for PIOC is more in males (69.3%) and occurs predominantly in the mandible (7:1 ratio compared to the maxilla). Maxillary lesions are typically anterior. Diagnosis requires ruling out oral mucosa carcinoma, antral primary tumors, and metastases. PIOC in the mandible usually develops above the inferior alveolar canal, while cyst-related cases are more common in the mandible. Many people use Waldron and Mustoe's classification, despite its exclusion of some newer types. Differentiating PIOSCC from maxillary sinus tumors, metastases, and alveolar cancers poses a challenge in diagnosis [4]. It can be hard to distinguish PIOSCC from primary tumors in the maxillary sinus or nasal lining, harmful ameloblastoma, oral mucosal SCCs, other tooth-related cancers, and cancers that have spread to the jaws [5,6].

## Case presentation

 A 46-year-old woman presented to the Department of Oral and Maxillofacial Surgery at our institute with complaints of pain, numbness in the left side of her lower lip, and reduced mouth opening for the past four months. She had a tobacco-chewing habit three to five times daily for 10 years, which she quit three months ago. Her family history and health conditions did not play a role. The patient reported visiting a local dental clinic in August 2024 related to pain and swelling in the left posterior mandible. A panoramic radiograph revealed a badly decaying second molar and a periapical infection associated with the left third molar. Based on the clinical and radiographic diagnosis, we carried out an extraction. However, the patient's persistent discomfort and unhealed socket following the extraction led to the start of an antibiotic prescription. Our center received the patient's referral due to the unresolved pain.

**Figure 1:**
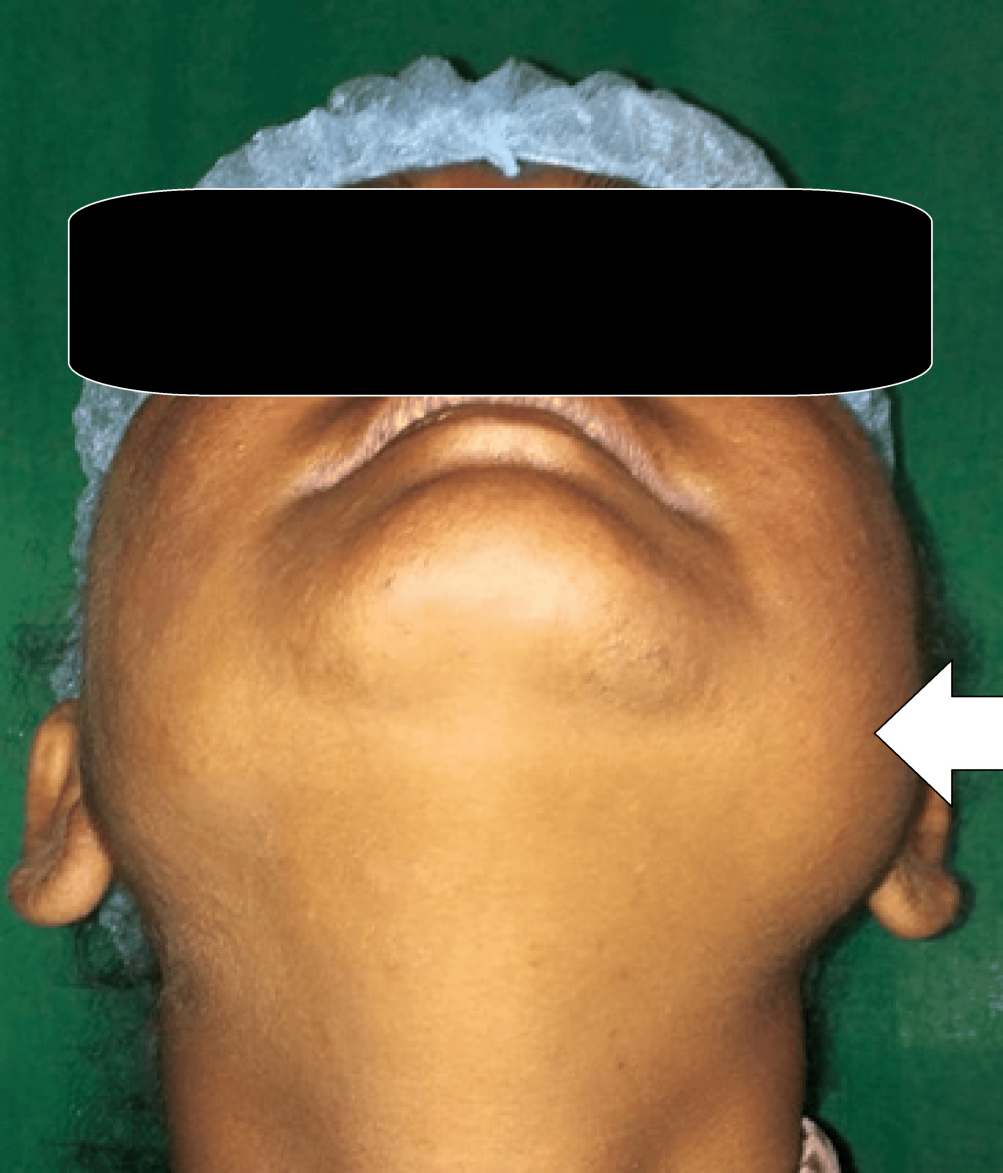
Preoperative images showing extraoral swelling on the left side of the face The arrow depicts the extraoral swelling on the left side of the face.

During the extraoral examination, the left side of the jaw showed a lot of bone growth and numbness in the lower lip, causing facial asymmetry. The patient had restricted mouth opening (interincisal distance <20 mm). The lymph node in the left submandibular region was tender on palpation, fixed, and palpable. There was no intraoral soft tissue tumor or ulceration seen during the clinical examination. However, palpation revealed an enlargement of the left ramus. Erythema was noted extending from the distal aspect of tooth 36 to the pterygomandibular raphe, with tenderness on palpation. On the alveolar ridge in the retromolar region, there was a sinus opening. Clinically, teeth 37 and 38 were absent (Figure 1).

**Figure 2:**
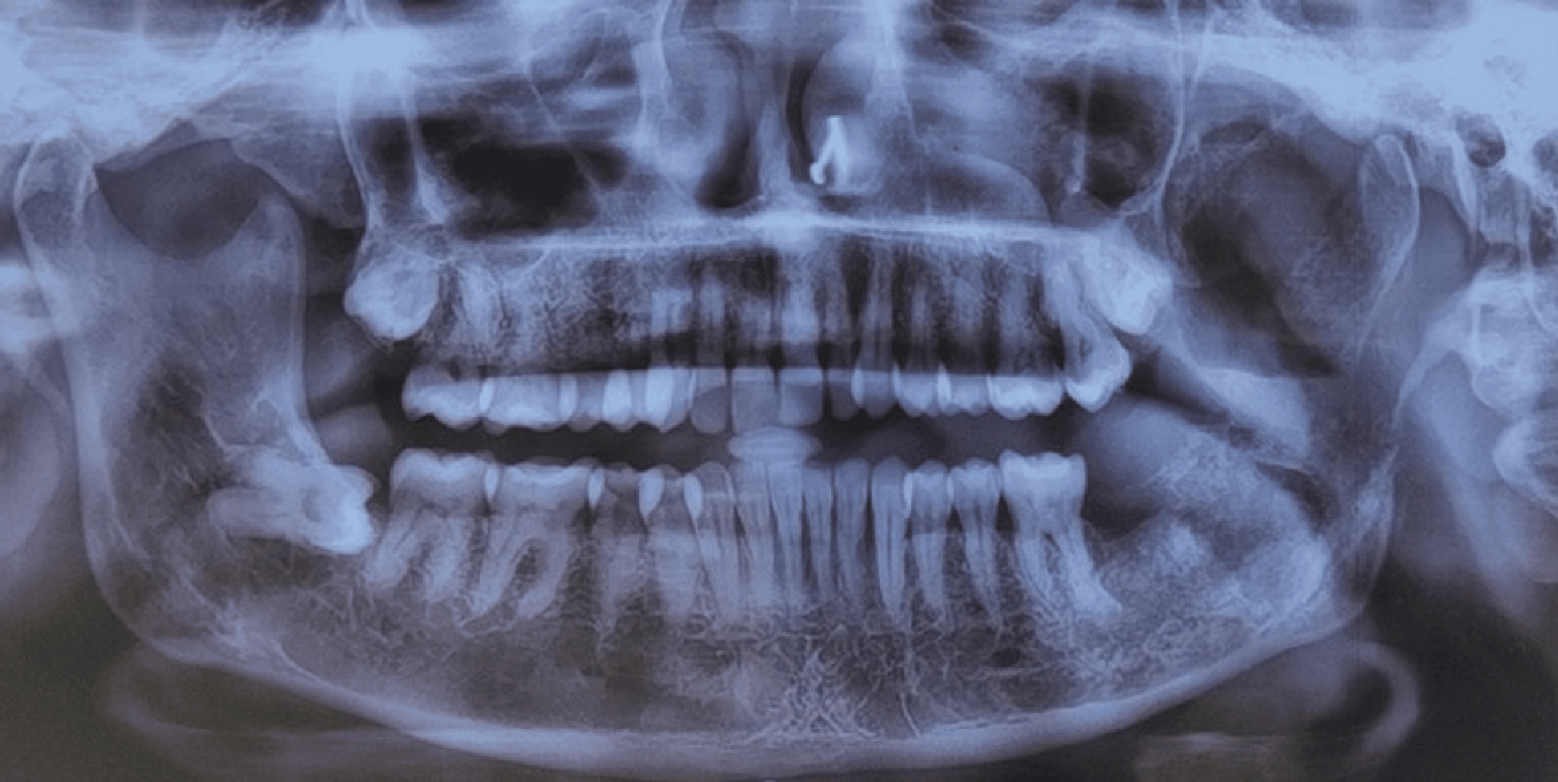
Orthopantomogram four months after the extraction of 37 and 38

For a preliminary diagnosis of an infective swelling, clinicians considered osteomyelitis, benign cysts, and tumors as differential diagnoses. After the patient's radiologic examination, an orthopantomogram showed that the left mandibular ramus had a radiolucent osteolytic lesion. With uneven, jagged edges, the lesion extended from the sigmoid notch to 5 mm above the lower border and from the ramus's anterior border to 3 mm before the posterior border. Although not clearly defined within the lesional area, the shape of the inferior dental canal was apparent (Figure 2).

**Figure 3:**
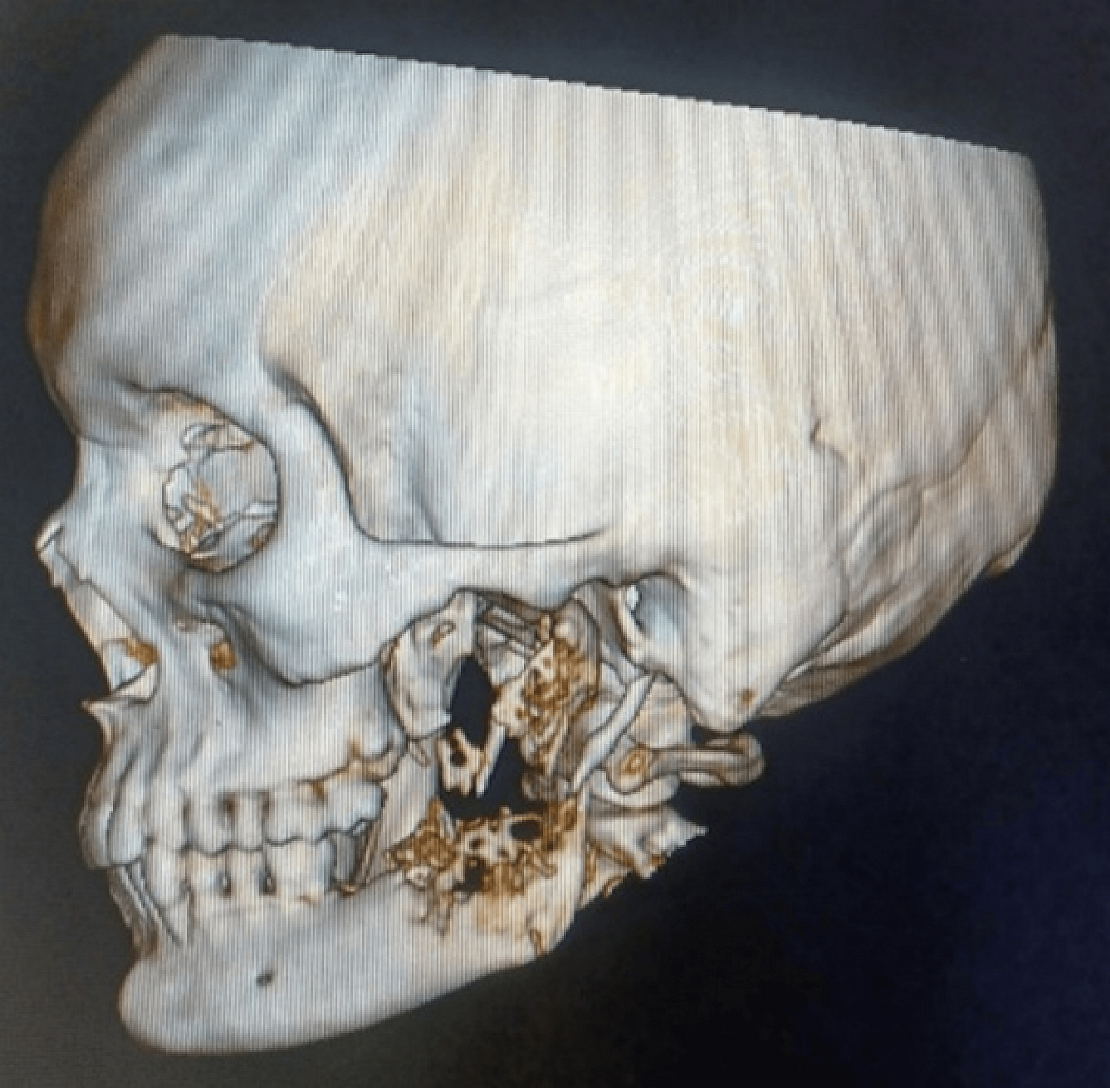
3D CT face showing the extent of the lesion

A 3D CT scan of the patient's face revealed an osteolytic lesion that extended from tooth 37 to 3 mm from the ramus's posterior border. Superoinferiorly, it involved the coronoid process and extended 5 mm from the mandibular lower border to the sigmoid notch level. The lesion had a characteristic "moth-eaten" appearance due to its uneven, hazy edges. Tiny particles of radiopacity, believed to be pieces of broken bone, were visible within the lesion. The lesion made the front edge of the ramus thinner and damaged the outer and inner layers of the ramus and the area around the third molar, with the inner layer being more affected. There was no lesion associated with the inferior alveolar nerve canal. Around the edges of the lesion, periosteal response and surrounding bone sclerosis were observed (Figure 3).

Based on history, clinical and radiological findings, a provisional diagnosis of chronic suppurative osteomyelitis was made. The following differential diagnoses were also considered.

Odontogenic carcinoma (ameloblastoma): Metastasizing ameloblastoma, ameloblastic carcinoma, solid-type PIOSCC, and those derived from keratocystic odontogenic tumors or cysts were among the differential diagnoses. This was due to the lesion's destructive nature, characterized by ill-defined, ragged borders on radiography. However, osteomyelitis was also considered because the patient had a history of tooth extraction, pus coming out, and X-ray signs such as periosteal reaction and hardening of the surrounding bone, which are common in osteomyelitis.

Metastatic carcinoma: Because the patient did not provide a history of the initial tumor elsewhere in the body, metastatic carcinoma was considered a differential diagnosis rather than a provisional diagnosis.

Sarcomas: Osteosarcoma and chondrosarcoma were also among the various differential diagnoses. Chondrosarcomas are rare in the jaws; they usually affect the mandibular angle, alveolar ridge, and anterior maxilla. In their early stages, they are painless and grow slowly. Only 7% of jaw cases are osteosarcomas, which are more common in long bones. In the osteolytic stage, they grow quickly and resemble moths. Tooth 33 is the average age of occurrence. Since the sunray appearance and Codman's triangle, seen in 25% of osteosarcoma cases, were not present in the X-ray results, they were not considered as initial diagnoses.

Odontogenic cyst and odontogenic tumor: Due to their radiological appearance of ill-defined radiolucency with ragged borders, these lesions, which are the most prevalent in the jaws, especially in the posterior region of the mandible, were ranked last in the differential diagnosis.

**Figure 4:**
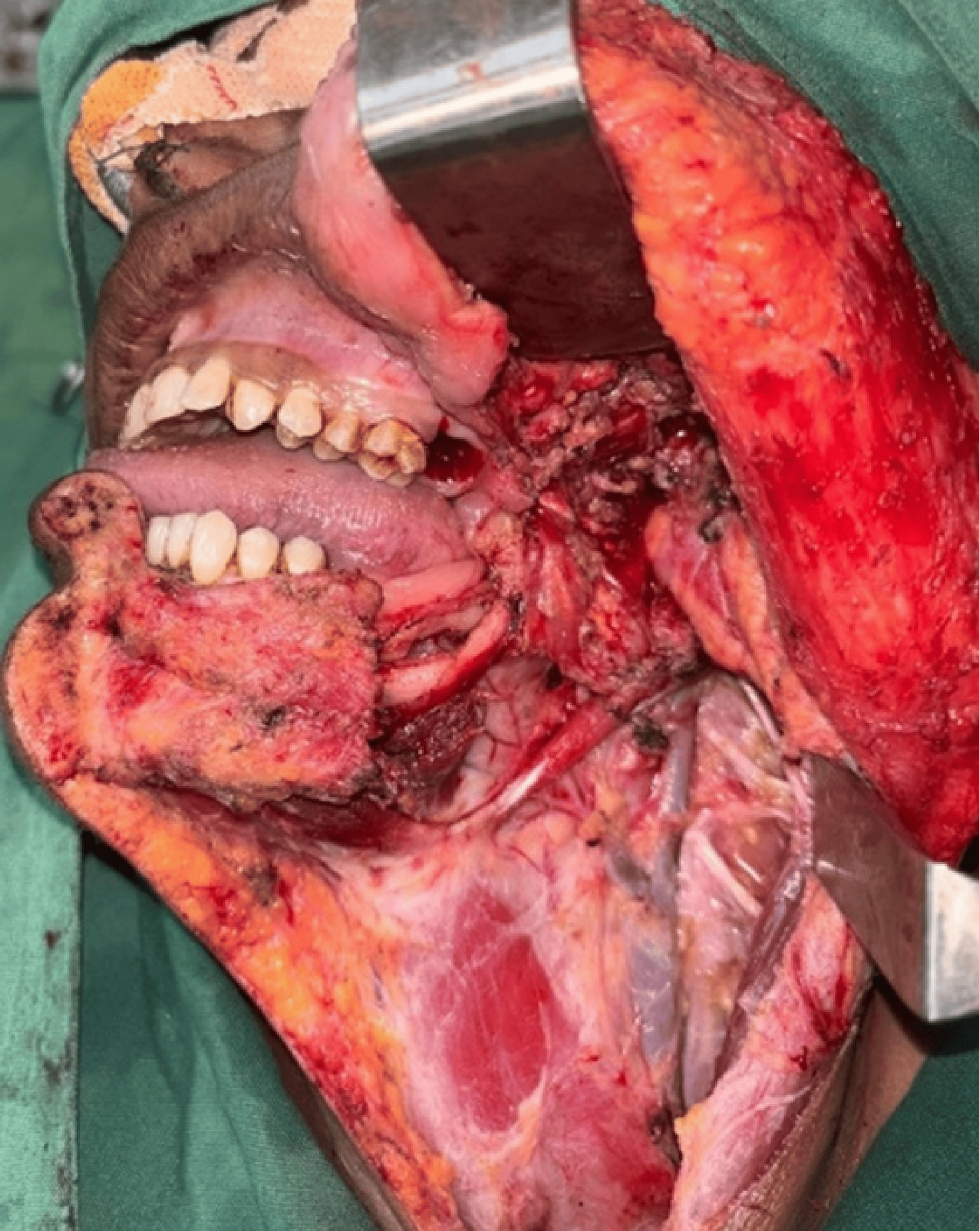
Segmental mandibulectomy where the left mandible distal to the 36 region till the condyle is removed and sent for frozen section for free margins, followed by left supraomohyoid neck dissection

Preoperative investigations included orthopantomography and 3D CT imaging, which revealed an ill-defined osteolytic lesion. A provisional diagnosis of chronic suppurative osteomyelitis was made. Blood tests before the surgery were normal. During the intraoperative phase, a segmental mandibulectomy was planned based on radiological aggressiveness. A frozen section biopsy was performed intraoperatively to obtain a rapid histopathological diagnosis, confirming well-differentiated squamous cell carcinoma inside the left mandible. Although no overt soft tissue lesion was clinically visible, the bony involvement permitted access for tissue sampling directly from the intraosseous lesion. We excised a 2.5 cm tumor that had penetrated 10 mm into the surrounding soft tissue and sent it for HP analysis. The surgical plan also included a contingency for neck dissection, to be carried out if malignancy was confirmed. Following intraoperative confirmation via frozen section, a left supra-omohyoid neck dissection was performed during the same surgical session (Figure 4).

**Figure 5:**
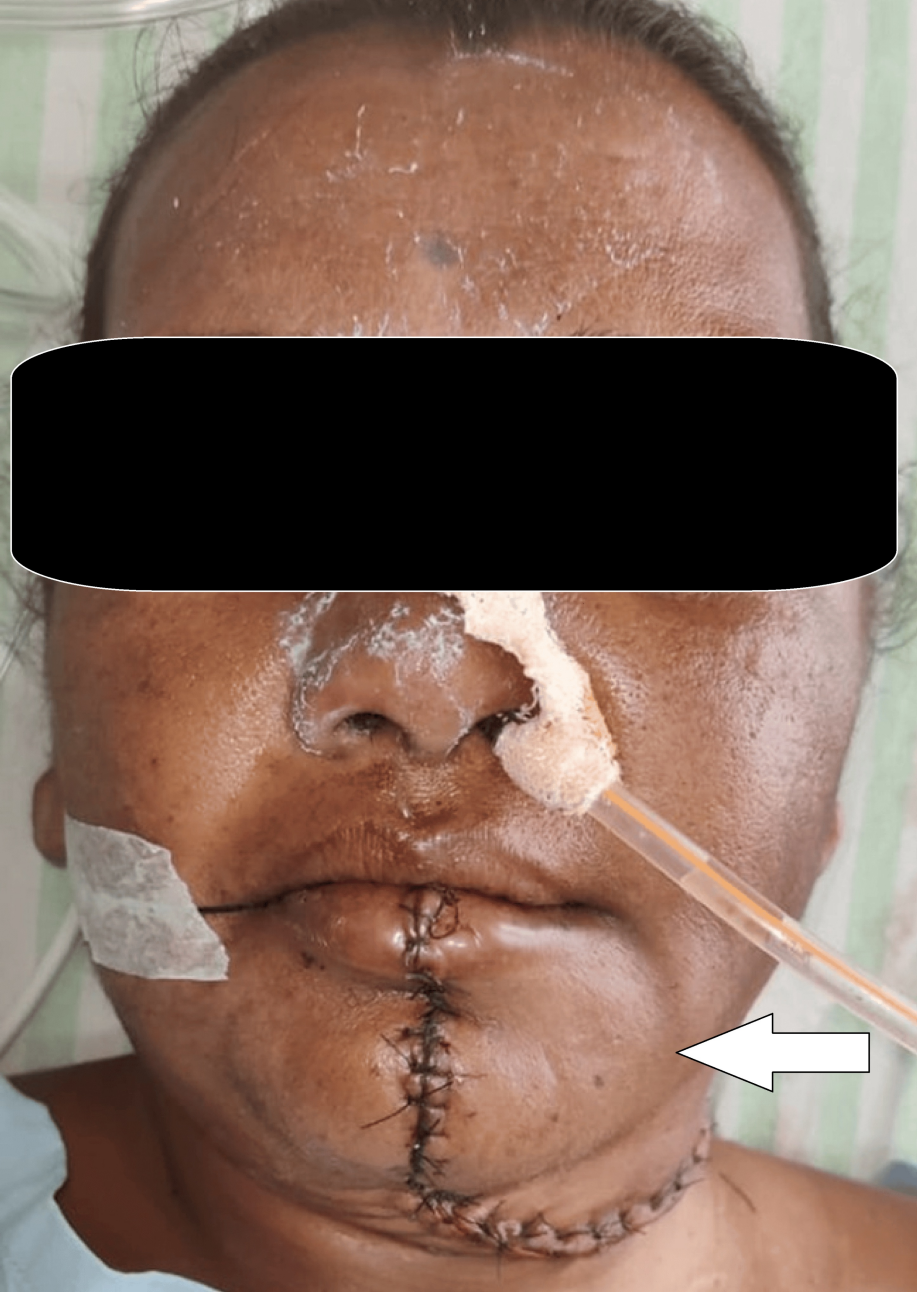
Extra-oral and intra-oral closure using synthetic bioabsorbable sutures 3-0 Vicryl and synthetic non-absorbable monofilament suture 2-0 Ethilon The arrow depicts post-operative reduction of the swelling on the left side of the face.

We cleaned the area where the surgery was done with a 10% betadine solution mixed with normal saline and then put in a Romo Vac drain and closed the layers using 3-0 Vicryl synthetic stitches that dissolve and 2-0 Ethilon synthetic stitches that do not dissolve (Figure 5).

**Figure 6:**
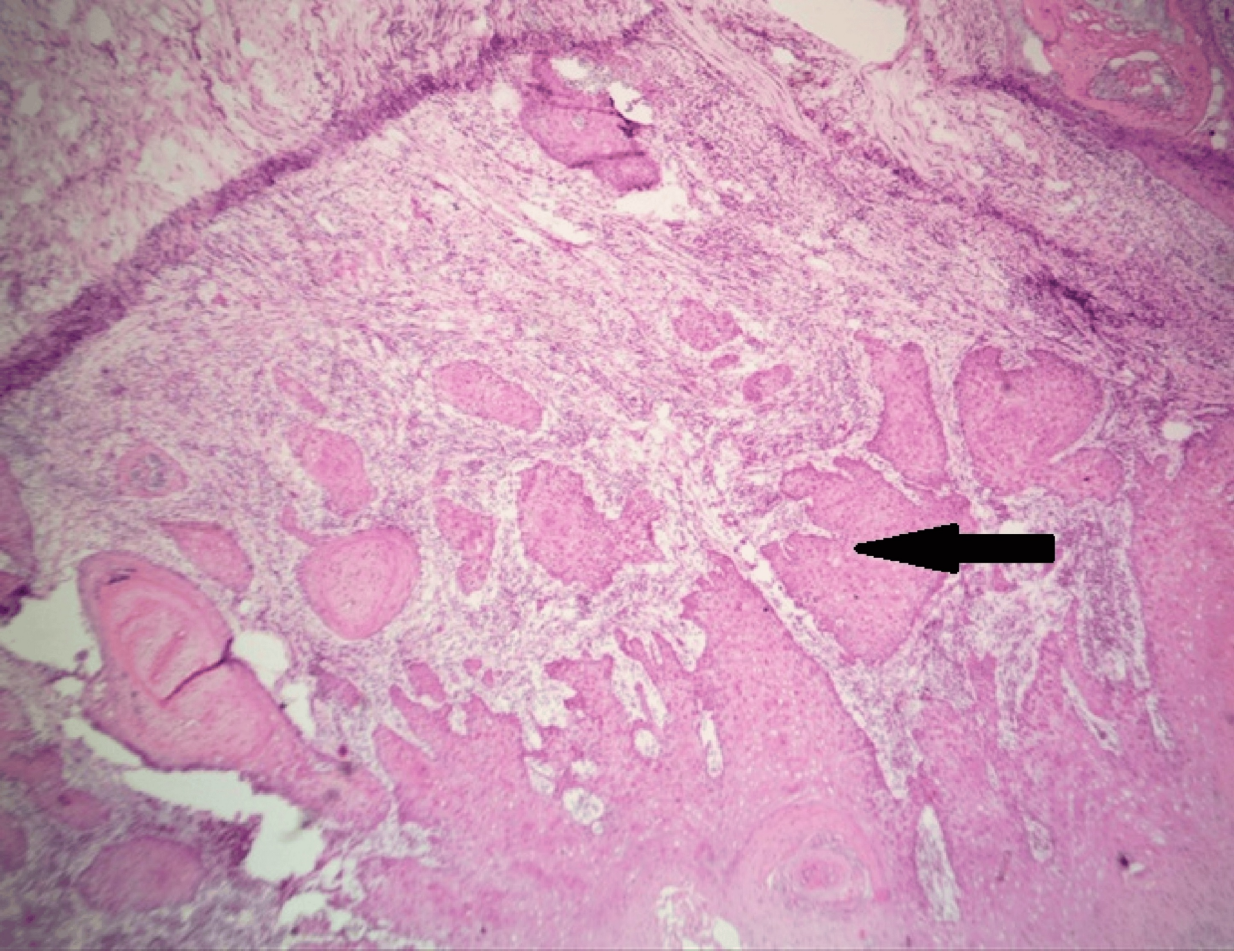
Microscopic image of frozen section showing intraosseous well-differentiated squamous cell carcinoma The arrow depicts the foci of the necrotic tissue. (H & E x40 – Scanner view)

The examination of the H&E-stained slides showed that there was a type of cancer called well-differentiated SCC inside the bone, which had many keratin pearls. Certain areas also displayed foci of tumor necrosis. The tumor had spots where it invaded the mandibular nerve both inside and around it, showing the most severe invasion pattern (type 5) (Figure 6).

**Figure 7:**
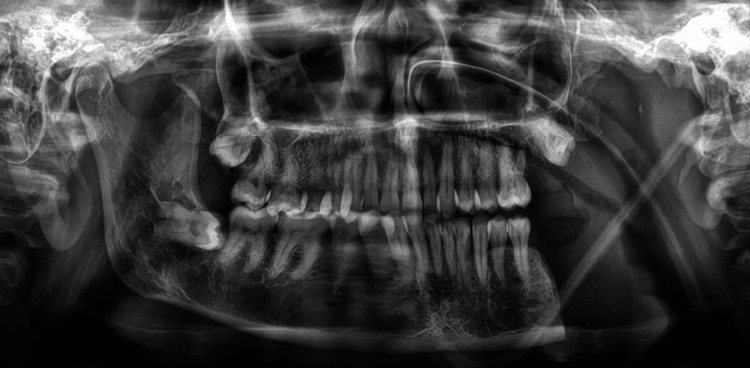
Post-operative radiograph

The American Joint Committee on Cancer (AJCC) staging system assigned a pathological TNM stage pT4a, pN0 to the tumor. Based on the microscopic results, we made a definitive diagnosis of PIOSCC of the left mandible-solid type. Postoperative healing was uneventful. Following hemimandibulectomy, the patient was planned for delayed mandibular reconstruction using a patient-specific titanium prosthesis and a potential fibula free flap. A guiding prosthesis was advised to minimize mandibular deviation and maintain occlusion. Future rehabilitation included implant-supported prosthodontics after oncological clearance (Figure 7).

## Discussion

PIOSCC is a rare malignant odontogenic tumor, accounting for 1%-2.5% of all odontogenic tumors. Although it can happen at any age, the fifth decade of life is when it most frequently occurs. The posterior mandible and mandibular body typically exhibit PIOSCC, not the maxilla [7]. Depending on its location, size, and nature, PIOSCC can present with various symptoms [8]. These may include common oral issues, discomfort, edema, and sensory abnormalities. Pain and edema are typical clinical manifestations of PIOSCC. In a review of 33 cases from around the world by Thomas et al., the most common symptom was pain, reported by 17 patients (54.8%), followed by jaw swelling in 16 patients (51.6%) and sensory issues in five patients (16.1%) [9].

Each form of PIOSCC has different radiographic characteristics. While other types of PIOSCC often look like harmless jaw problems with clear borders at first, solid-type PIOSCC appears as a bone-destroying area with unclear, rough edges. Larger PIOSCCs seriously damage the jawbone [2]. In terms of radiological and clinical features, PIOSCC is comparable to odontogenic tumors [10]. In the early stages, PIOSCC can sometimes look like common dental problems, such as gum disease or issues around the tooth root, which might lead to a late or wrong diagnosis. In their examination of 24 PIOSCC patients, Kaffe et al. discovered that 13% were mixed radiolucent-radiopaque (all in the mandible) and 87% were radiolucent. Additionally, 56.5% had defined non-corticated boundaries, 43.5% had diffuse borders, and none had corticated borders [11]. In our case, radiolucency and the removed bone fragments gave the lesion a heterogeneous appearance. The boundaries were noncorticated and poorly delineated [12].

Under a microscope, PIOSCC can range from poorly formed non-keratinizing cancers to well-formed tumors with a lot of keratin. It might show a tooth-related pattern with basal-type cells creating small spaces or a layered structure with cells lined up at the edges. It can show a tooth-related pattern with basal-type cells forming small sacs or a woven structure with cells lined up at the edges. These cells frequently have their nucleus oriented opposite to the basement membrane. When identifying different types of harmful tooth-related tumors, such as malignant calcifying epithelial odontogenic tumor, ameloblastic carcinoma, central mucoepidermoid carcinoma, odontogenic ghost cell carcinoma, and clear cell odontogenic carcinoma, it is important to consider PIOSCC. Focal points of central necrosis or degeneration may also be visible within the epithelial islands [13].

Three criteria were established by Suei et al. [14] to diagnose PIOC as no surface mucosal ulcers, unless brought on by injury or dental extraction; a histologic examination must show SCC free of cystic components or other odontogenic tumor cells in order to rule out other odontogenic carcinomas; and chest radiographs must be clear upon diagnosis and for at least six months thereafter in order to rule out a distant primary tumor. In our case, we made the diagnosis of PIOSCC (formerly known as PIOC de novo keratinizing type) after considering the aforementioned parameters. It can show a tooth-related pattern with basal-type cells forming small sacs or a woven structure with cells lined up at the edges. It is radical surgery. According to some writers, chemotherapy alone is unsuccessful in cases where lymph nodes are involved; thus, block resection along with the removal of the main tumor is advised, followed by radiation and chemotherapy. Postoperative adjuvant chemotherapy or radiation therapy is still unclear and needs more research [5]. Because of the small number of documented instances, different treatment modalities, and irregular follow-up times, it is difficult to predict the prognosis for patients with PIOSCC. The five-year survival rate for patients with conventional OSCC is 57.6% for those over 40 and 66.2% for those under 40. On the other hand, PIOSCC has a worse prognosis than typical OSCC, with a five-year survival rate ranging from 30% to 46%. Improving the prognosis of these uncommon malignancies requires prompt detection and treatment [13].

## Conclusions

Histopathologically, PIOSCC can be confused with other intraosseous carcinomas, especially ameloblastic carcinoma. It is a rare tumor with a poor prognosis. Intraosseous carcinomas should be taken into consideration when a patient exhibits chronic discomfort, edema, and an intraosseous osteolytic lesion with irregular boundaries. To establish the diagnosis of PIOSCC, other intraosseous carcinomas must be ruled out.
